# Application of transcranial direct current stimulation in cricopharyngeal dysfunction with swallowing apraxia caused by stroke: A case report

**DOI:** 10.1097/MD.0000000000027906

**Published:** 2021-12-03

**Authors:** Juan Yang, Huozhong Yuan

**Affiliations:** Master of Medicine, Ganzhou People's Hospital, China.

**Keywords:** balloon dilatation, cricopharyngeal dysfunction, stroke, swallowing apraxia, transcranial direct current stimulation

## Abstract

**Rationale:**

Dysphagia is a common complication after stroke. The 2 types of dysphagia with cricopharyngeal dysfunction and swallowing apraxia after stroke are relatively rare and difficult to treat; however, there are few clinical case reports of cricopharyngeal dysfunction and swallowing apraxia after stroke.

**Patient concerns:**

A case of cricopharyngeal dysfunction and swallowing apraxia due to cerebral infarction caused by atrial fibrillation in a 63-year-old woman who was followed up for 1 year.

**Diagnoses:**

The patient was diagnosed with cricopharyngeal dysfunction and swallowing apraxia caused by stroke based on the clinical course and imaging findings.

**Interventions:**

Pharmacotherapy and rehabilitation therapy.

**Outcome:**

The patient's swallowing function returned to normal, and her nasal feeding tubes were removed, and oral feeding was resumed.

**Lessons:**

The 2 types of dysphagia with cricopharyngeal dysfunction and swallowing apraxia after stroke are relatively rare and difficult to treat after stroke. Only by improving swallowing apraxia can patients perform mandatory swallowing and balloon dilatation treatment. However, transcranial direct current stimulation has a good therapeutic effect on the primary motor and sensory cortex of the tongue in patients with cricopharyngeal dysfunction and swallowing apraxia.

## 1 Introduction

Dysphagia is a common complication after stroke and exhibits different symptoms because of the different stroke sites. The manifestations of cricopharyngeal dysfunction caused by medullary infarction include upper esophageal sphincter contraction, relaxation, and coordination dysfunction and the decrease of the contractile force of the pharyngeal constrictor, lack of larynx lift, residual, infiltration, and aspiration in the epiglottis valley and pyriform sinus.^[1]^ Swallowing apraxia is characterized by uncoordinated lip, tongue, and mandible movements without sensory impairment or dyskinesia during oral swallowing. The patient's automatic and unconscious swallowing function is largely preserved,^[2]^ but the absence of tongue movement or a significant decrease in the range of motion during autonomous and conscious swallowing results in delayed initiation of food delivery.^[3]^ It is rare for patients to suffer from cricopharyngeal dysfunction and dysphagia caused by stroke. Dou et al^[4]^ have used active balloon dilatation therapy to treat cricopharyngeal dysfunction and have achieved a good effect, but active balloon dilatation therapy requires patients to follow the swallowing instructions of doctors or rehabilitation therapists to achieve this effect. When patients suffer from swallowing apraxia, it is difficult to carry out swallowing instructions with active balloon dilatation therapy and basic deglutition training, which greatly increases the difficulty of treatment. Currently, there are limited clinical treatments for swallowing apraxia, and we present a rare case comparing the efficacy of transcranial direct current stimulation (tDCS) before and after treatment.

## 2 Case presentation

### 2.1 Patient data

**Figure 1:**
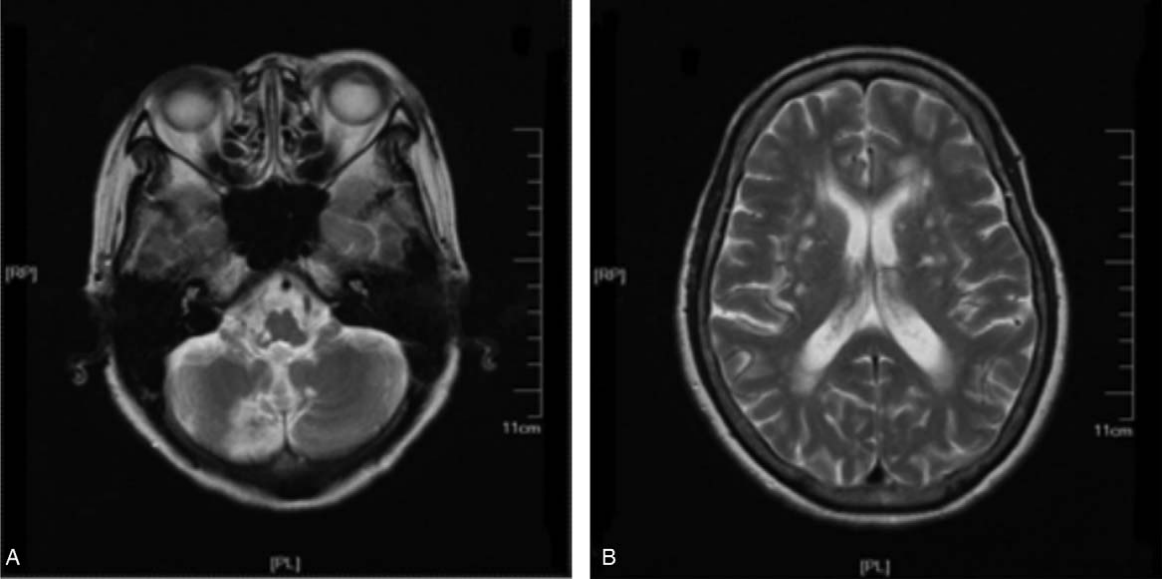
Brain MRI of the case showed patchy acute infarction in the right medulla oblongata and the right cerebellar hemisphere (A), multiple small patches of ischemia, infarction, and softening lesion also occurred in the pons, beneath the bilateral frontoparietal cortex, centrum semiovale, usually at the side of the lateral ventricle and in the basal ganglia (B). MRI = magnetic resonance imaging.

The patient was a 63-year-old woman. She was right-handed, and she was hospitalized mainly because of “dizziness, walking instability with having difficulty in eating for 3 days.” When admitted to the hospital, she could not consume any food through her mouth. Physical examination showed that the patient had clear consciousness, poor mental state, unclear articulation, and compared with the left side, a relatively weaker ability for the right soft palatal lift and absent pharyngeal reflex. Cranial magnetic resonance imaging showed patchy acute infarction in the right medulla oblongata and right cerebellar hemisphere. Multiple small patches of ischemia, infarction, and softening lesions were located in the pons, beneath the bilateral frontoparietal cortex, centrum semiovale, mostly at the side of the lateral ventricle and in the basal ganglia (Fig. 1 A, B). Videofluoroscopic swallowing study (VFSS) showed that the cricopharyngeus muscle opened partly when carrying out reflex swallowing and could not complete swallowing commands. Clinical diagnoses include cerebral infarction, swallowing apraxia, cricopharyngeal dysfunction, dysarthria, and atrial fibrillation.

### 2.2 Therapies

The patient was successively treated with therapy A (basic swallowing training and balloon dilatation) for 4 weeks and therapy B (tDCS, basic swallowing training, and balloon dilatation) for 4 weeks. The specific treatment therapies used were as follows.

#### 2.2.1 tDCS treatment

IS200 type transcranial direct current stimulator from Sichuan, China was used, and doctors chose a 6.0 × 4.2 cm^2^ isotonic saline gelatine sponge electrode as the stimulating electrode. And the projection area of the labial cortex is located under the Cz point of the International electromyogram (EMG) 10 to 20 System 75 mm, the upper and lower 2.5 mm is the area where the maximum activation site of the sensorimotor cortex is in the left hemisphere during lip tongue pronunciation.^[5]^ When treating, the patient was seated, the anode was placed at a location point on the body surface of the labial tongue cortex (as mentioned above), and the cathode was placed on the contralateral shoulder. The treatment was conducted alternately on the left and right sides for 15 min each with a 30-minute interval. The electrical stimulation intensity was 1.2 mA (current density was approximately 50 μA/cm^2^). Treatment was performed once a day, 5 days per week.

2.2.2 Basic swallowing training includes breathing training, neck movement training, oral and facial muscle group movement training, sensory training, tongue movement training, swallowing coordination training, vocal cord adduction training, laryngeal up-lift training, empty swallowing and forced empty swallowing training, cough reflex training, and low-frequency electric stimulation treatment. Low-frequency electric stimulation treatment used a Vitalstim low-frequency electric therapy apparatus from the United States. When treating, the patient's head remained neutral. The 1 and 2 electrodes of channel 1 were close to the hyoid above the horizontal arrangement. The 3 and 4 electrodes of channel 2 were located along the midline of the anterior portion. Among these electrodes, the 3 electrodes were placed in the top-notch of the thyroid, and the 4 electrodes were placed below the thyroid notch. The treatment parameters were as follows: bidirectional square wave, pulse width 300 μS, frequency of electrical stimulation 80 Hz, on/off ratio 300:100, and stimulus intensity 0 to 25 mA. If the swallowing muscles in the neck of the patients were vibratory accompanied by swallowing movements, and patients could tolerate it, we considered it as the criterion. Each treatment lasted for 20 min. The basic swallowing training was performed once a day, 5 days per week.

#### 2.2.2 Balloon dilatation treatment was as follows

Active balloon dilatation treatment was used for treatment. First, a 14 latex double-lumen catheter with the nasal esophagus is inserted, ensuring that the catheter goes down into the esophagus and completely through the cricopharyngeus muscle, and water was infused (water temperature approximately 0°C) in the catheter, approximately 6 to 8 mL, making the balloon diameter expand from 20 to 25 mm. Next, the catheter is slowly pulled outwards until it feels stuck or cannot be moved, indicating that the balloon has arrived at the lower margin of the circumpharyngeal muscle, and it should be tagged; then, an appropriate amount of ice water is drawn out (according to the tension of the circumpharyngeal muscle, if the catheter can pass it when the balloon is pulled out, we think it is appropriate), and the catheter is gently pulled out again and again. The patient was asked to swallow the balloon actively while the balloon was pulled upward. Once the patient has a feeling of slipping and the resistance decreases sharply, the ice water in the balloon should be pulled out quickly and repeated 8 to 10 times. Notably, when the pull-out resistance decreases sharply, it indicates that the balloon is outside the front part of the larynx, and the fluid in the balloon must be quickly evacuated to avoid suffocation. The balloon dilatation treatment was performed once a day for 30 minutes, 5 days per week.

### 2.3 Evaluation methods

Before the treatment, after 4 weeks of therapy A and after 4 weeks of therapy B, 2 uniformly trained and qualified swallowing and speech therapists evaluated the efficacy of the therapy, including the following items.

2.3.1 Tongue movement and facial-oral apraxia were assessed using the psycholinguistic assessment in Chinese aphasia version 1.0 was used to evaluate lip closure, mouth opening, laryngeal elevation, tongue movement, and facial-oral apraxia. The score criteria for facial-oral apraxia were as follows: inability to perform (imitation) counts, 0 points; slight performance (imitation) counts, 1 point; performs (imitation) counts slightly worse, 2 points; and performs normal (imitation) counts, 3 points.^[6]^

2.3.2 Examination of swallowing function using VFSS was as follows: considering that barium is not easily absorbed due to malabsorption, we selected 76% meglumine diatrizoate and 50% glucose as the contrast agent, according to a ratio of 2:1, and used this to prepare different types of food in the following proportions with nutritive rice flour, including thin liquid (30 mL contrast agent and 3 g nutrition rice flour), thick liquid (30 mL contrast agent and 8 g nutrition rice flour), and paste meal (30 mL contrast agent and 14 g nutrition rice flour). Doctors observed the movement of food (1, 2, and 4 mL of food were given in turn and quantified by syringes and spoons) through the mouth cavity, pharynx, and esophagus when patients completed reflex swallowing (automatic and unconscious swallowing) and voluntary or command swallowing (the operator gives patients food through the mouth with a spoon and asks them not to swallow for a while then immediately to complete the swallowing after the operator gives the verbal command “swallow”) and observed whether the contrast agent remained in the epiglottis valley and piriform foss, regardless of whether there was aspiration and whether the cricopharyngeus muscle was open. If the patient had aspirated by mistake, the angiography would be stopped, and the angiography should be discharged in time. The oral stage scoring criteria were as follows: food cannot be transported from the mouth cavity to the pharynx, food will be out of the mouth or by food gravity into the pharynx, 0 score counts; patients are unable to stir food into a mass and transport it to the throat, food enters the pharynx in fragments, 1 score count; some food remains in the mouth after 1 swallow, 2 score counts; food can be completely in the pharynx after 1 swallow; and 3 point counts. The pharynx and larynx stage scoring standards were as follows: 0 points indicate decreased swallowing reflex, poor closure of laryngeal elevation, and soft palatal arch elevation; 1 point means that there is considerable food remaining in the epiglottis valley and piriform foss; 2 points means that there is a small amount of food remaining in the epiglottis valley and piriform foss, and all remaining food can be swallowed into the throat after repeated swallowing; and 3 points means food can enter the esophagus completely after swallowing. The esophageal stage score standard was as follows: 0 points, a large number of misabsoption and no cough occurred; 1 point means a large number of misabsoption and cough occurred; 2 points means a small number of misabsoption and no cough occurred; 3 points means a small amount of misabsoption and cough occurred; and 4 points means no misabsoption and no cough occurred.^[7]^

## 3 Results

The patient experienced any maladjustment or intolerance during treatment, and after 4 weeks of treatment with therapy A, the patient's tongue movement and facial-oral apraxia had no improvement; after 4 weeks of treatment with therapy B, the tongue movement of the 2 patients was significantly improved, and the facial-oral apraxia score increased from 10 to 42 points. VFSS examination showed that the patient could perform mandatory swallowing, and she could complete food agitation and transportation when swallowing autonomously. There was no delay in the swallowing reflex, the laryngeal elevation was close to normal, and the cricopharyngeus muscle could open in a coordinated manner. Food is able to smoothly enter the esophagus and stomach. Thus, her nasal feeding tubes were removed, and oral feeding was resumed. The treatment results are presented in Tables 1–3.

**Table 1:** Analysis of the results of tongue movement.

Inspection time	Tongue position	Unconscious movement	Command execution	Feeding test
On admission	Slight retraction	Reach outside the teeth for 0.5 cm	Cannot	Cannot
After 4 weeks of therapy A	Slight retraction	Reach outside the teeth for 0.5 cm	Cannot	Cannot
After 4 weeks of therapy B	Slight retraction	Reach outside the teeth for 2 cm	Can reach outside the teeth for 2 cm	Can

**Table 2:** Analysis of examination results of the patient with oral and facial apraxia.

	Cough		Nasal breathe		Blow out a match		Blow a straw		Blow a drum cheek		Pout	
Inspection time	Perform	Imitation	Perform	Imitation	Perform	Imitation	Perform	Imitation	Perform	Imitation	Perform	Imitation
On admission	0	2	0	1	0	0	0	1	0	1	0	1
After 4 weeks of therapy A	0	2	0	1	0	0	0	1	0	1	0	1
After 4 weeks of therapy B	1	2	1	2	2	2	1	2	1	2	2	3

	Close lips		Show teeth		Stick out tongue		Open mouth		Throat		
Inspection time	Perform	Imitation	Perform	Imitation	Perform	Imitation	Perform	Imitation	Perform	Imitation	Total score
On admission	0	1	0	1	0	1	0	1	0	0	10
After 4 weeks of therapy A	0	1	0	1	0	1	0	1	0	0	10
After 4 weeks of therapy B	2	2	1	2	2	3	2	3	2	2	42

**Table 3:** Analysis of VFSS scores.

	Oral period		Throat period		Esophageal period		
Inspection time	Reflective	Mandatory	Reflective	Mandatory	Reflective	Mandatory	Total score
On admission	2	Cannot perform	2	Cannot perform	1	Cannot perform	5
After 4 weeks of therapy A	2	Cannot perform	2	Cannot perform	1	Cannot perform	5
After 4 weeks of therapy B	3	Can perform	3	Can perform	4	Can perform	10

## 4 Discussion

Human swallowing reflex activity, whose neural control consists of 3 parts, is very complex: afferent and efferent systems are composed of cranial nerves, the brainstem swallowing center, and a higher cortical swallowing center.^[8,9]^ After stroke, the cortex, cortical brainstem tracts or the brainstem, and the kernel of the medulla oblongata will become diseased, and the brainstem deglutition center regulation mechanism is abnormal, which may easily lead to dysfunction of the lower jaw, lip, tongue, soft palate, pharynx, cricopharyngeus muscle, and esophagus, eventually affecting the patient's deglutition function.^[8]^ At present, it is believed that dysphagia is related to lesions in the cerebral hemispheric cortex^[3]^ or periventricular white matter.^[10]^ Yuan et al^[11]^ used EMG to record the changes in EMG activity in 6 normal subjects and 1 patient with swallowing apraxia after cerebral infarction in 3 states: quiet closed eyes, reflex swallowing, and voluntary swallowing. It was observed that when patients were swallowing voluntarily, the excitability of the left central, parietal, and posterior temporal cerebral cortex was lower than that of quiet closed eyes swallowing and reflex swallowing. Related functional magnetic resonance imaging studies have shown that the lateral surface of the anterior and posterior gyri are the most common activation areas of the swallowing cortex in normal subjects, while activation was also observed in the forehead, cingulate gyrus, parietooccipital region, and temporal lobe.^[12,13]^ In this study, the patient had a stroke induced by atrial fibrillation, whose lesions involved the medulla oblongata and cerebral cortex. The patient also developed cricopharyngeal dysfunction and swallowing apraxia, which is consistent with the above findings.

Relevant studies have found that the central nervous system has strong plasticity and functional reorganization ability after stroke, which can achieve functional improvement through repeated training. tDCS is a non-invasive transcranial stimulation technique that can regulate cortical excitability through microcurrents, whose stimulation effect has polarity specificity, such as anodic stimulation that will lead to depolarization of resting membrane potential and increase cortical excitability, which leads to hyperpolarization of the resting membrane potential and decreases cortical excitability.^[14]^ Yuan et al^[11]^ applied electro-encephalography (EMG) to observe the changes in cortical excitability of patients with swallowing apraxia caused by stroke, and found that the excitability of the swallowing cortex significantly increased in patients with significant improvement in swallowing apraxia after treatment with tDCS. Lang et al^[15]^ used tDCS to stimulate the left M1 region and used single-pulse transcranial magnetic stimulation to evaluate the potential amplitude, onset latency, and transcallosal inhibition time evoked by contralateral movement, which indicated that tDCS not only affects corticospinal circuits involved in the generation of motor-evoked potential but can also inhibit transcallosal regulation of the interneurons to the contralateral hemisphere. Considering the above contralateral inhibitory factors, tDCS was used for patients with cricopharyngeal dysfunction and swallowing apraxia to stimulate the primary motor and sensory cortex of the tongue in the bilateral brain. It was found that after tDCS treatment, tongue movement and orofacial apraxia were significantly improved in the patient, and her scores were significantly improved (from 10 to 42 points). VFSS examination after 4 weeks of therapy B treatment showed that the patient could perform mandatory swallowing, and she could complete food agitation and transportation when swallowing autonomously. There was no delay in swallowing reflex, the laryngeal elevation was close to normal, and the cricopharyngeus muscle was able to open in a coordinated manner. Food was able to smoothly enter the esophagus and stomach. Therefore, her nasal feeding tube was removed, and oral feeding was resumed. Yuan et al^[16]^ applied tDCS to stimulate the bilateral primary cortex of the swallowing sensation and motor in patients with swallowing apraxia directly. These researchers observed that the swallowing apraxia symptoms of patients after treatment were significantly improved in both voluntary and reflex swallowing. The results of the EEG examination also suggested that the excitability of broad areas of the swallowing cortex was improved, which was consistent with the results of this study.

In conclusion, the results of this study show that tDCS stimulation has a good therapeutic effect on the primary motor and sensory cortex of the tongue in patients with cricopharyngeal dysfunction and swallowing apraxia caused by stroke. Only by improving swallowing apraxia can patients perform mandatory swallowing. By further participating in basic swallowing training and active balloon dilatation therapy, patients can achieve satisfactory rehabilitation of swallowing disorders. This combined therapy warrants further study and development.

## Acknowledgments

We would like to thank the members and staff of the Department of Rehabilitation Medicine of the Ganzhou People's Hospital who contributed to this case.

## Author contributions

JY contributed to the data collection and writing. HY guided the completion of the study. All authors contributed to manuscript revision and read and approved the submitted version.

**Project administration:** Huozhong Yuan.

**Writing – review & editing:** Juan Yang.
